# *Klebsiella pneumoniae* arms itself: poultry food chain drives spread and evolution of *mcr-1.26-*IncX4 plasmids

**DOI:** 10.1128/spectrum.04210-23

**Published:** 2024-05-01

**Authors:** Ulrike Binsker, Claudia Jäckel, Jörg Rau, Maria Borowiak, Carina Salzinger, Isidro García-Meniño, Annemarie Käsbohrer, Jens André Hammerl

**Affiliations:** 1 Department for Biological Safety, German Federal Institute for Risk Assessment, Berlin, Germany; 2 Chemical and Veterinary Analysis Agency Stuttgart (CVUAS), Fellbach, Germany; 3 Laboratorio de Referencia de Escherichia coli (LREC), Dpto. de Microbioloxía e Parasitoloxía, Universidade de Santiago de Compostela (USC), Lugo, Spain; 4 Instituto de Investigación Sanitaria de Santiago de Compostela (IDIS), Santiago, Spain; 5 Department for Farm Animals and Veterinary Public Health, Unit of Veterinary Public Health and Epidemiology, University of Veterinary Medicine Vienna, Vienna, Austria; Instituto de Higiene, Montevideo, Uruguay

**Keywords:** *K. pneumoniae*, colistin, *mcr*, antimicrobial resistance, livestock, transmission, plasmid, genome

## LETTER

Colistin resistance is primarily driven by the acquisition of the mobile colistin resistance gene (*mcr*) through horizontal gene transfer (1). *mcr-1* is the most widespread *mcr* determinant globally and appears in 36 variants with varying frequencies (2). Rare *mcr* variants are instrumental in tracing the epidemiology of these genes and associated mobile genetic elements. Recently, the rare variant *mcr-1.26* has been identified in 16 colistin-resistant extended-spectrum β-lactamase (ESBL)-producing and commensal *Escherichia coli* from poultry and a human clinical isolate in Germany (3, 4). Bioinformatic analysis revealed that these isolates shared a distant relationship but carried *mcr-1.26* on highly similar IncX4 plasmids. This finding signified the establishment and dissemination of *mcr-1.26*-IncX4 plasmids within the poultry food chain and its transmission to humans.

**Fig 1:**
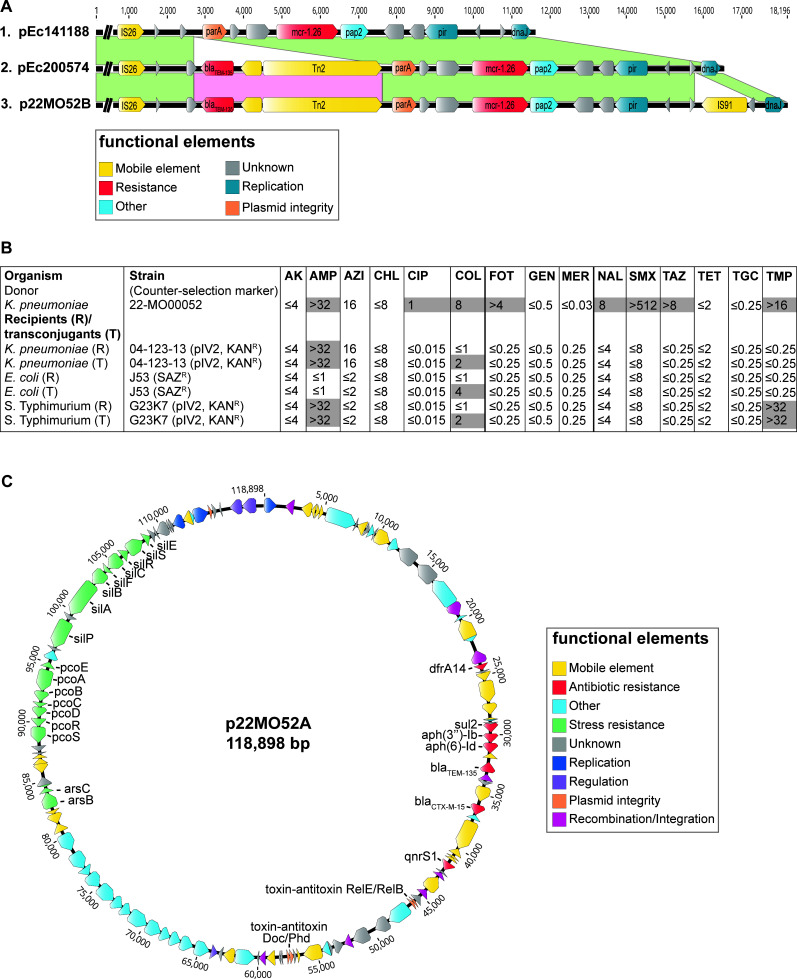
Organization and interspecies transmission of resistance plasmids. (**A**) Comparison of the *mcr-1.26*-containing region of IncX4 plasmids between *E. coli* and *K. pneumoniae* isolated from poultry. (**B**) Interspecies transmission by conjugation of IncX4 plasmid and resulting phenotypic colistin resistance in transconjugants. Microbiological resistance profiles were determined using the broth microdilution method according to CLSI. Resistances in donor strain 22-MO00052, recipient strains (R), and corresponding transconjugants (T) are highlighted with a gray background. AK, amikacin; AMP, ampicillin; AZI, azithromycin; FOT, cefotaxime; TAZ, ceftazidime; CHL, chloramphenicol; CIP, ciprofloxacin; COL, colistin; GEN, gentamicin; MERO, meropenem; NAL, nalidixic acid; SMX, sulfamethoxazole; TET, tetracycline; TGC, tigecycline; and TMP, trimethoprim. (**C**) Schematic representation of the IncFIB plasmid p22MO52A.

Here, we report for the first time the presence of an *mcr-1.26*-IncX4 plasmid in a *Klebsiella pneumoniae* isolate 22-MO00052 (CVUAS 34108) obtained from pre-packaged raw turkey meat in Germany in 2022. Hybrid whole-genome sequencing (Illumina/ONT, Bioproject PRJNA1038782) revealed the location of *mcr-1.26* on a 39.953-kb IncX4 plasmid (p22MO52B). The plasmid also carried the Tn*2*-associated beta-lactamase gene *bla*_TEM-135_ (99.88% nucleotide identity), but acquired additionally an IS91 family transposase distinguishing it from pEc200574 (Fig. 1A; Table 1) (3). A BlastN search showed that the IS91 family transposase is primarily found on plasmids of the *Enterobacteriaceae* family, associated with antibiotic resistance and virulence genes, creating a potential hotspot for acquiring additional pathogenicity genes (5, 6). p22MO52B exemplifies the ongoing evolution and adaption of *mcr-1.26*-IncX4 plasmids.

**TABLE 1:** Genetic features of *K. pneumoniae* 22-MO00052 and its plasmids*^a^^,^^b^*

Feature	Chromosome	Plasmids	
ID	22-MO00052	p22MO52A	p22MO52B
Size (kb)	5,347.601	118.898	39.953
G + C content (%)	57.24	50.83	43.36
Molecule type	Circular	Circular (IncFIB, 98.93%)	Circular (IncX4, 100%)
Accession number	CP138466	CP138467	CP138468
ORFs^*e*^	4,956	111	42
Resistance determinants	+**^*e*^**	+	+
Aminoglycosides	–^*f*^	*aph* (6)-Id (100%)^*c*^	-
	–	*aph*(3″)-Ib (100%)^*c*^	–
Beta-lactams	*bla*_SHV-27_ (100%)^*c*^	–	–
	–	*bla*_TEM-135_ (99.88%)^*c*^	*bla*_TEM-135_ (99.88%)^*c*^
	–	*bla*_CTX-M-15_ (100%)^*c*^	–
Carbapenems	*ompK*37 (I70M),^*d*^ *ompK*37 (I128K)^*d*^	–	–
Cephalosporins	*ompK*36 (N49S),^*d*^ *ompK*36 (L59V),^*d*^ *ompK*36 (T184P)^*d*^	*–*	–
Colistin	–	–	*mcr*-1.26 (100%)^*c*^
Disinfectants	*oqxA* (99.40%)^*c*^	–	*–*
	*oqxB* (99.14%)^*c*^	–	*–*
Fosfomycin	*fosA* (99.76%)^*c*^	–	*–*
Quinolone	*–*	*qnrS*1 (100%)^*c*^	*–*
Fluoroquinolone	*acrR* (P161R), *acrR* (G164A), *acrR* (F172S), *acrR* (R173G), *acrR* (L195V), *acrR* (F197I), *acrR* (K201M)	*–*	*–*
Sulphonamides	*–*	*sul*2 (100%)^*c*^	*–*
Trimethoprim	*–*	*dfrA*14 (100%)^*c*^	*–*
IS-elements			
Insertion sequence	ISKpn1, IS100, IS911, ISEc30, ISEc52, ISKpn1	ISKpn19, ISEc52, ISKpn26, IS26	IS26, IS91
Composite transposon	n.d.^*e*^	cn_5504_IS26	n.d.
Unit transposon	n.d.	n.d.	Tn*2*

^*a*^ Nucleotide sequence identity is given for some elements in brackets.

^*b*^ The *K. pneumoniae* strain 22-MO00052 harbored three additional plasmids that did not carry resistance genes: p22MO52C [accession: CP138469, Col RNAI (89.26%), 5.631 kb, 47.38% GC, six ORFs], p22MO52D [accession: CP138470, Col440I (97.37%), 3.631 kb, 44.20% GC, three ORFs], and p22MO52E [accession: CP138471, Col MG828 (94.27%), 2.967 kb, 52.85% GC, two ORFs].

^*c*^ Acquired resistance determinants.

^*d*^ Mutations are predicted to play a role in phenotypic carbapenem and cephalosporin resistance.

^*e*^ n.d., not detected; +, present; and ORF, open reading frame.

^*f*^ "-" in the Table means "not present".

Filter mating experiments of p22MO52B using *K. pneumoniae* 22-MO00052 as a donor demonstrated an *mcr-1.26* transfer frequency of 4.7 × 10^2^ to *E. coli*, 2.3 × 10^1^ to *K. pneumoniae*, and 1.1 × 10^3^ to *Salmonella enterica* Typhimurium, confirming its potential for interspecies transmission (Fig. 1B). While the notification of *mcr* genes in klebsiellae is not new, studies focusing on emerging *mcr* variants, specifically *mcr-1.26*, have provided valuable insights into the origin and dynamics of *mcr*-associated colistin resistance development. *mcr-1.26* serves as a suitable indicator to shed light on the spread of resistance genes across various bacterial hosts and One Health compartments.

In addition to p22MO52B, the *K. pneumoniae* isolates carried a 118.898-kb multidrug-resistance plasmid p22MO52A belonging to the incompatibility group IncFIB (Fig. 1C). p22MO52A had no similarity to any other plasmid in the NCBI database (accessed 18 October 2023) and carried a 24.4-kb integron with seven resistance genes, conferring resistance to four classes of antibiotics (Table 1). The integron contained a second *bla*_TEM-135_ gene (99.88% nucleotide identity), but unlike p22MO52B, this gene was not linked to Tn2. The integron was associated with an ISKpn19 element and was identical to the integrons of the IncY and IncFIB plasmids from *E. coli* (LR999865.1, host *Branta leucopsis*) and *K. pneumoniae* (CP084503, host *Capra aegagrus hircus*), respectively (accessed 13 November 2023).

The isolate belongs to ST716 [KL 110 (unknown capsule type, not serologically defined), O1/O2v1 (O2a O-type)], which has been associated with human infections (Pathogenwatch, https://pathogen.watch/, accessed 07 November 2023). In addition to the acquired resistance genes, 22-MO00052 carried chromosomal mutations in *ompK*36, *ompK*37, and *acrR*, which are predicted to contribute to carbapenem, cephalosporin, and fluoroquinolone resistance, respectively (Table 1) (7–10). Besides carbapenem, the isolate exhibited phenotypic resistance toward cephalosporins and fluoroquinolones, which could be mediated by both chromosomal mutations and acquired resistance genes (Fig. 1B). *K. pneumoniae* is an [ESKAPE(E)] microorganism, known for its excessive exchange of genetic information with the environment and other bacteria, including *mcr-1.26*, as an adaptation to varying selective pressures in different ecosystems (11). *K. pneumoniae* is also a nosocomial pathogen for which novel therapeutic approaches are needed, as recognized by the World Health Organization.
